# Correlations between weight-bearing 3D bone architecture and dynamic plantar pressure measurements in the diabetic foot

**DOI:** 10.1186/s13047-020-00431-x

**Published:** 2020-10-30

**Authors:** Claudio Belvedere, Claudia Giacomozzi, Claudio Carrara, Giada Lullini, Paolo Caravaggi, Lisa Berti, Giulio Marchesini, Luca Baccolini, Stefano Durante, Alberto Leardini

**Affiliations:** 1 Movement Analysis Laboratory, IRCCS Istituto Ortopedico Rizzoli, Bologna, Italy; 2 Department of Cardiovascular, Endocrine-Metabolic Diseases and Aging, Istituto Superiore di Sanità (Italian National Institute of Health), Viale Regina Elena 299, 00161 Rome, Italy; 3 Department of Medical and Surgical Sciences, “Alma Mater” University, Bologna, Italy; 4 Nursing, Technical and Rehabilitation Assistance Service, IRCCS Istituto Ortopedico Rizzoli, Bologna, Italy

**Keywords:** Foot bone models, Bone positions and orientations, Cone-beam weight-bearing computed tomography, Principal component analysis, Diabetic foot, Dynamic plantar loading

## Abstract

**Background:**

Measurements of plantar loading reveal foot-to-floor interaction during activity, but information on bone architecture cannot be derived. Recently, cone-beam computer tomography (CBCT) has given visual access to skeletal structures in weight-bearing. The combination of the two measures has the potential to improve clinical understanding and prevention of diabetic foot ulcers. This study explores the correlations between static 3D bone alignment and dynamic plantar loading.

**Methods:**

Sixteen patients with diabetes were enrolled (group ALL): 15 type 1 with (N, 7) and without (D, 8) diabetic neuropathy, and 1 with latent autoimmune diabetes. CBCT foot scans were taken in single-leg upright posture. 3D bone models were obtained by image segmentation and aligned in a foot anatomical reference frame. Absolute inclination and relative orientation angles and heights of the bones were calculated. Pressure patterns were also acquired during barefoot level walking at self-selected speed, from which regional peak pressure and absolute and normalised pressure-time integral were worked out at hallux and at first, central and fifth metatarsals (LOAD variables) as averaged over five trials. Correlations with 3D alignments were searched also with arch index, contact time, age, BMI, years of disease and a neuropathy-related variable.

**Results:**

Lateral and 3D angles showed the highest percentage of significant (*p* < 0.05) correlations with LOAD. These were weak-to-moderate in the ALL group, moderate-to-strong in N and D. LOAD under the central metatarsals showed moderate-to-strong correlation with plantarflexion of the 2nd and 3rd phalanxes in ALL and N. LOAD at the hallux increased with plantarflexion at the 3rd phalanx in ALL, at 1st phalanx in N and at 5th phalanx in D. Arch index correlated with 1st phalanx plantarflexion in ALL and D; contact time showed strong correlation with 2nd and 3rd metatarsals and with 4th phalanx dorsiflexion in D.

**Conclusion:**

These preliminary original measures reveal that alteration of plantar dynamic loading patterns can be accounted for peculiar structural changes of foot bones. Load under the central metatarsal heads were correlated more with inclination of the corresponding phalanxes than metatarsals. Further analyses shall detect to which extent variables play a role in the many group-specific correlations.

## Background

Diabetes is a pandemic, with a forecast of up to 600 million patients all over the world by 2045 [1]. Particularly, complications of the foot represent one of the most common, costly and severe complications of this metabolic disease [1]. In 2018 prevalence of diabetic foot complications ranged from 3.3% (Australia) to about 15% (South America) [2, 3], and a very recent review paper showed that the economic burden on the patients ranges, on average, from the equivalent of 6-day income in the United States to about 6-year income in India, i.e. the worst-case scenario [4].

Knowledge and prevention of diabetic foot disorders are thus important and may be improved by biomechanical measurements in real patients. The literature has long reported static and dynamic plantar loading assessments alone, but more recent studies have proposed integration with kinematics measurements [5], especially for a more accurate analysis of plantar loading under specific and well detected foot regions [6, 7]. Recently, the cone-beam computed tomography (CBCT) technology has given access to three-dimensional (3D) measures of bone alignment in weight-bearing condition [8–13], finally overcoming traditional 2D measurements from radiographs [14, 15] and standard computed tomography scans in supine position. Investigation of 3D orientation or misalignment of foot bones under physiological load has great potential for a better interpretation of the relevant plantar loading changes, which may expose the diabetic foot to a high risk of ulceration. In fact, bone orientation measurements in weight-bearing condition, both in absolute and relative terms, can now reveal the skeletal architecture in 3D, with the necessary accuracy and repeatability [16]. It has been also demonstrated that, with respect to standard computed tomography, radiation doses are smaller, device ergonomics and portability are better, and overall costs are smaller with the new technique [17–19] and these instruments are expected to be used extensively for clinical and biomechanical measurements of the foot in the next decades.

Only one recent study has reported data on the possible relationships between 3D bone orientation and plantar loading [20]. However, in this study measurements were taken with a plantar pressure plate positioned into the CBCT for simultaneous data collection, i.e. in up-right double leg posture in that device. In this static condition, no statistically significant correlations were found between 3D foot angles and plantar force and pressure.

The present study is primarily aimed at exploring possible correlations between foot bone position and orientation 3D measurements from CBCT scans, i.e., in static conditions, and regional plantar loading from plantar pressure measurements during barefoot level walking, i.e., in dynamic conditions. The main hypothesis is that changes in 3D forefoot architecture correlate with foot dynamic loading alterations. This is meant to contribute to the prevention of the risk of foot ulceration in patients with diabetes, finally understanding which foot bone abnormalities may have an impact on plantar pressure. The study is secondarily aimed at exploring possible correlations between the bone 3D measurements and the major functional, biological and clinical parameters, namely arch index and contact time, age, and neuropathy-related measures.

## Methods

Sixteen patients with diabetes performed two data acquisition sessions, the CBCT static scan and the plantar pressure analysis during gait. These patients were grouped as follows: 8 type 1 diabetes without (D) and 7 with (N) neuropathy, and 1 with latent autoimmune diabetes of the adults (LADA), all together in the ALL group. Those patients with two out of the following three conditions [21] were assigned to the neuropathic group N: Michigan Neuropathy Screening Instrument (MNSI) > 2, Michigan Diabetes Neuropathy Score > 7, and biothesiometer Vibration Perception Threshold (VPT) > 25 Volt. All patients signed an informed consent to the study, approved by the local Ethical Committee (Prot. IOR 7685 28th July 2017).

**Fig. 1:**
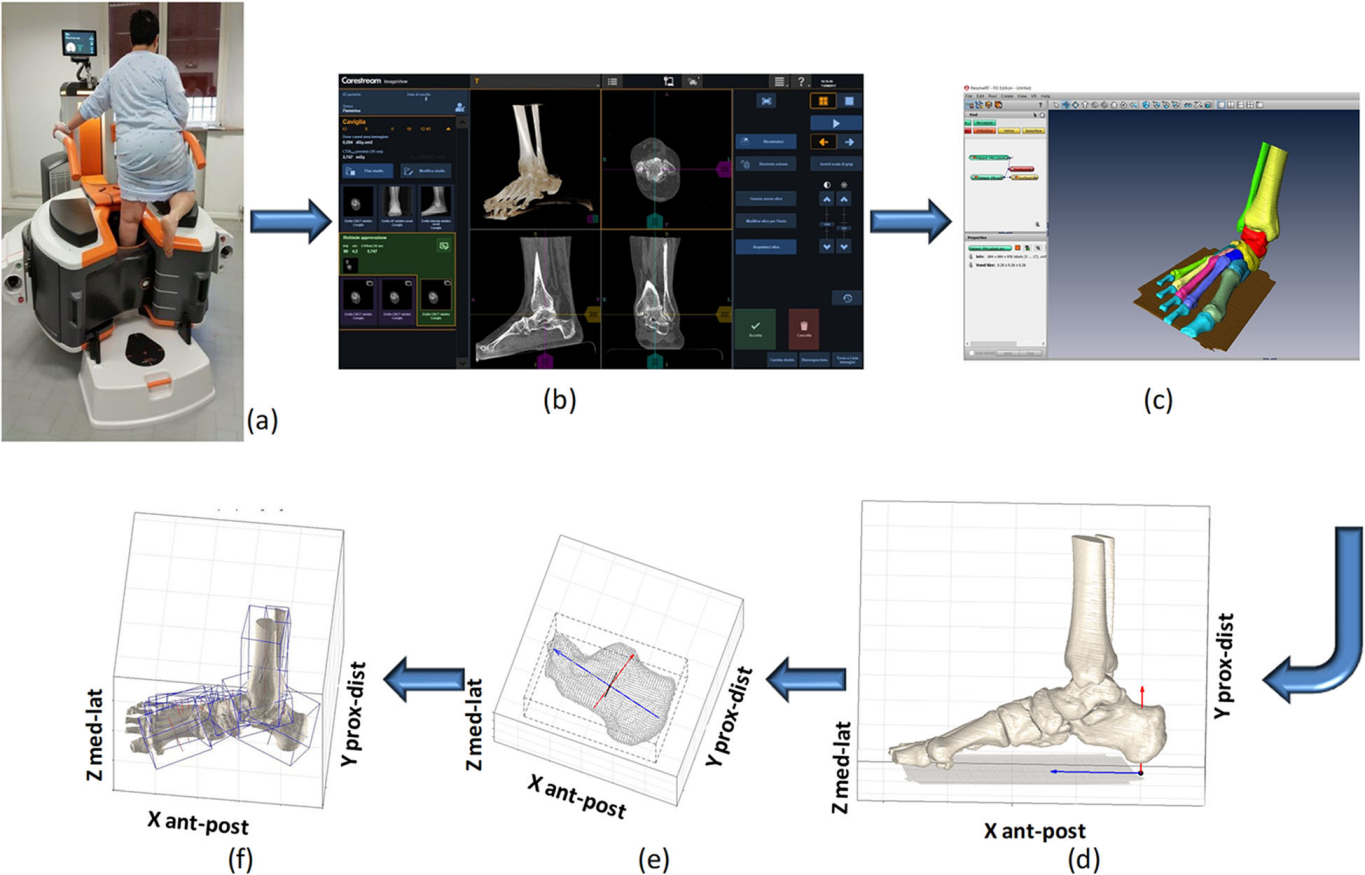
Pictures representing the process from CBCT scans to the embedded bone reference frames. Pictures representing the process from CBCT scans to the embedded bone reference frames, through the definitions of 3D models of the bone surface. The patient in single-leg weight-bearing during the CBCT scan (**a**). The 3D data-set including volume rendering available at the interactive screen (**b**). The result of the process of bone segmentation (Amira): all foot and ankle bone segments were modeled separately (different colors), and the ground segment is also identified and depicted (**c**). The overall bone models in the foot anatomical reference frame, here in a nearly lateral view (**d**). Construction of the three anatomical axes by means of the PCA technique, an exemplary application to the calcaneus model; the origin at the centroid of the bone model and the three corresponding co-ordinate axes are depicted (**e**). The same, for all foot bone models (**f**)

Both feet were scanned (‘OnSight 3D Extremity System’, Carestream, Rochester, NY;) in single-leg upright posture (Fig. 1a), with the instruction to the patient to put full load on the analyzed foot and to use the other contacts just for equilibrium [13]. This CBCT scan lasted about 20 s; 3D interactive rendering of the foot from automatic image processing were then obtained a few minutes later (Fig. 1b). In the present preliminary analysis only one, randomly selected foot was analyzed for each patient. For this foot scan, virtual slicing set at 0.26 mm distance was performed, which resulted in a standard DICOM file format. This file was processed in Amira™ (Thermo Fisher™ Scientific, Waltham, MA-USA), where semi-automatic segmentation of each bone was performed, resulting in corresponding 3D models in STL format (Fig. 1c). The ground was identified and segmented as well, and taken in the overall 3D reconstruction as the orientation of the transverse anatomical plane of the foot. These STL files were imported in Matlab® (Mathworks Inc., Natick, MA-USA), where the following analysis was performed, according to recently established techniques [22]. Because the foot bones and the ground were in their original technical frame of the CBCT device, thus not along the anatomical planes, a foot anatomical reference frame was first defined as follow: the vertical axis was orthogonal to the ground, the antero/posterior axis was the line segment on the ground plane joining the projections of the most plantar points of the calcaneus and of the second metatarsal head; these two axes define the lateral plane of the foot, and their cross product the medio-lateral axis. All bone segments were then realigned in this anatomical reference frame (Fig. 1d).

**Fig. 2:**
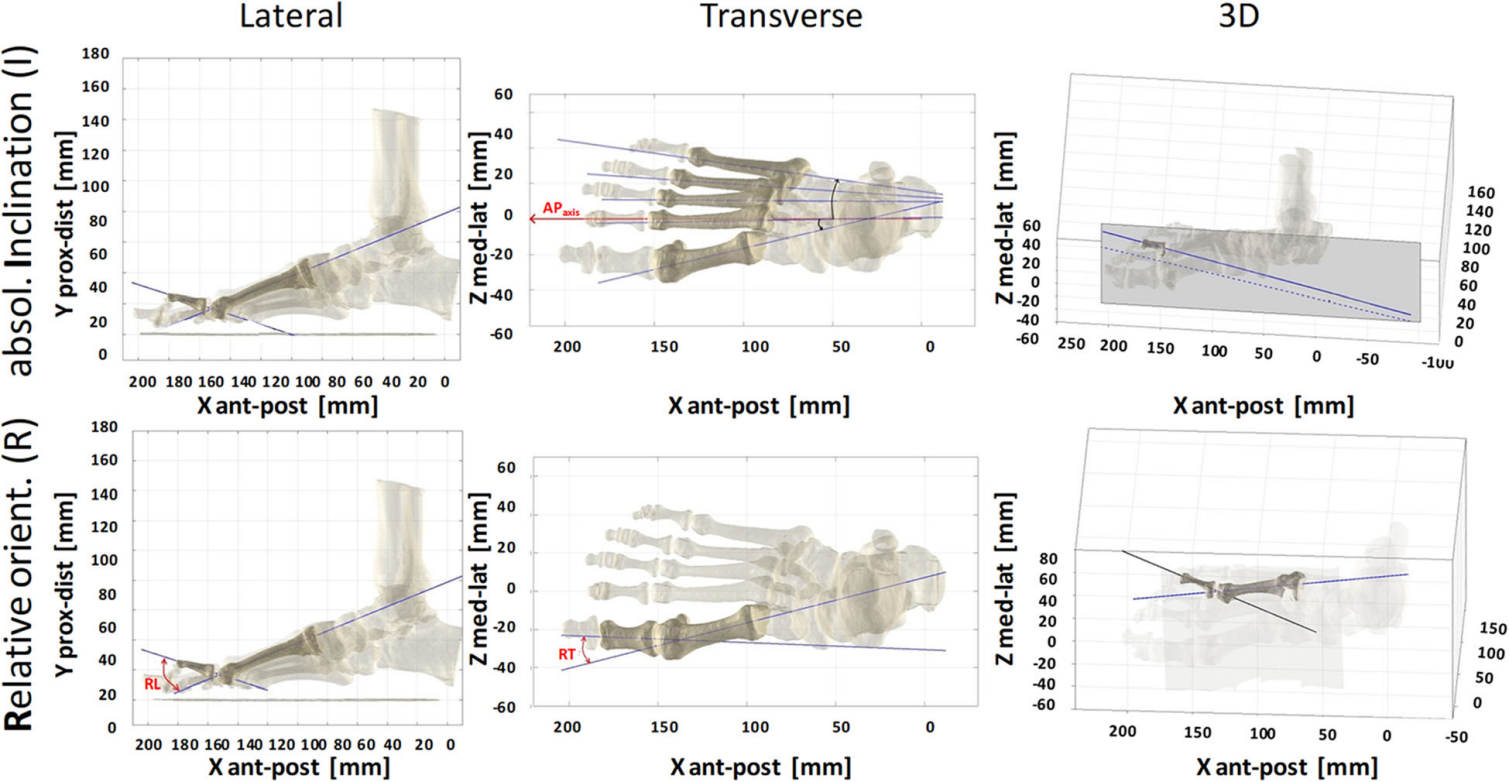
Diagrams from an exemplary foot model. Diagrams from an exemplary foot model of the absolute inclination (top row) and relative orientation (bottom), in the lateral (left column) and transverse (central) projections, and in 3D (right), according to the foot anatomical reference frame (see also Fig. 1d). Longitudinal axes of a few exemplary bones and their inclination and orientation angles are depicted

For each of these bone models, an anatomical reference frame was defined using the Principal Component Analysis (PCA) (Fig. 1e, f) [22]. This entails searching the three axes with the highest variance in the 3D coordinates of the bone surface points, under the constraint that these axes must be orthogonal to each other. Because of foot bones shape, this technique identifies the longitudinal, i.e. nearly antero/posterior, medio-lateral and dorsi-plantar anatomical directions. The advantage of this technique is represented by the automatic generation of these axes, in a one-shot calculation, without human, subjective intervention. The bone models were also projected into the lateral, frontal and transverse planes of the foot anatomical reference frame (Fig. 2), where the angles of absolute inclination (I), i.e., of the single bone, and relative orientation (R), i.e., between two adjacent bones, were calculated, similarly to what defined traditionally in foot radiographs [14, 15]. These bone embedded reference frames were ultimately oriented in the 3D foot reference frame, and the corresponding I and R angles were thus calculated both in 3D and in the Lateral, Frontal and Transverse anatomical planes, thus respectively denoted as: I3 (absolute 3D inclination), IL (absolute inclination in the Lateral plane), IF (absolute inclination in the Frontal plane), IT (absolute inclination in the Transverse plane), and R3 (relative 3D orientation), RL (relative orientation in the Lateral plane), RF (relative orientation in the Frontal plane), RT (relative orientation in the Transverse plane).

**Fig. 3:**
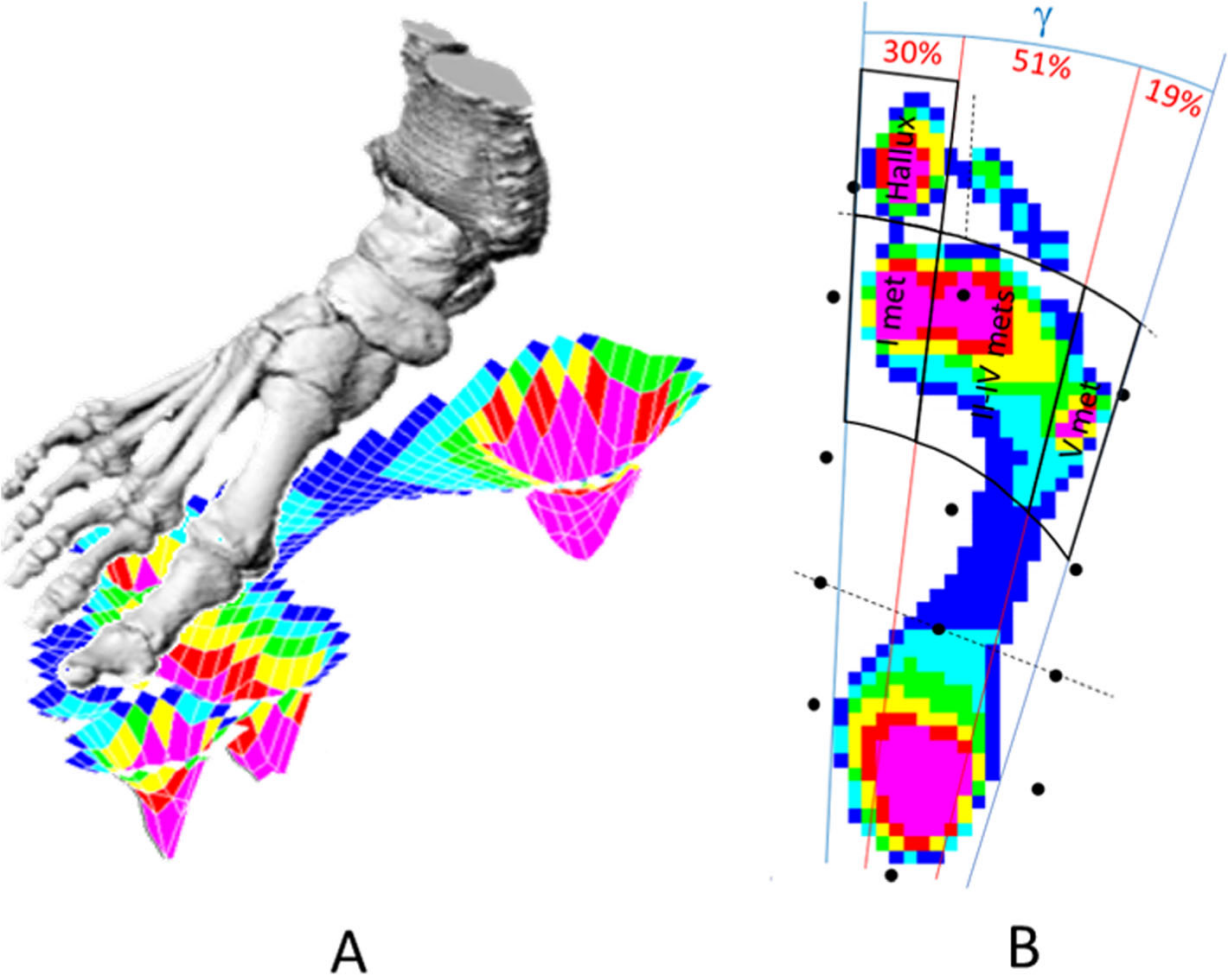
Diagrams representing the present measurements. **a** Diagrammatic representation in 3D of the combination of the full foot bone model (above) registered on the corresponding pressure footprint (below). **b** Subdivision of the footprint (from the same patient) in the four forefoot regions (thicker black lines); the overall angle of the footprint (γ) is divided in three arcs (red lines), and the projection of the anatomical markers of the foot are also shown (black points)

Pressure patterns were acquired during barefoot level walking at self-selected speed through a capacitive sensor platform (EMED® q-100, novel GmbH, Munich, Germany; 4 sensors / cm^2^; range 0–1270 kPa; 100 Hz) [23], synchronized with an eight-camera 3D motiontracking system (Vicon®, Oxford, UK) and previously integrated with the Rizzoli Foot Model [6], designed to track multi-segment foot kinematics. The platform was embedded flush in the middle of a long pathway. To capture full footprints of at-regimen steps and to avoid gait pattern modifications while targeting the platform, patients were asked and controlled to walk while looking straight ahead. Five such consistent full footprints were saved for each patient and foot, and these were then registered and averaged. The feet were instrumented with the established marker-set of the Rizzoli Foot Model [24]. Motion and pressure data collected for the same foot selected for the CBCT dataset underwent further analysis, in particular anatomical foot masking based on the anatomical skin markers, to detect forefoot and hallux regions [6]. To further split the forefoot into three relevant sub-regions, lines were drawn to divide the whole foot plantar angle (Fig. 3b), i.e. between the lateral and medial tangents of the footprint, into three angles equal to 30, 51 and 19% of the overall plantar angle, to define respectively I, II-to-IV and V metatarsal regions [25]. Proprietary EMED software (novel GmbH, Munich, Germany) and purposely developed Matlab codes were used for the whole data processing.

From 3D foot models obtained from the CBCT scans, in the present preliminary work only the I and R angles of the five metatarsal and phalanx bones (i.e. M1–5, P1–5), in 3D and in the lateral, frontal and transverse plane projections were analyzed, for a total of 60 measurements. In addition, the minimum height of these 10 bones from the ground were calculated as the minimum distance from the ground and expressed both in absolute terms and also relative to their minimum height, for a total of 20 additional measurements. Absolute and relative height of the cuboid and the navicular bone, to represent the longitudinal arch height, led to a total of 84 measurements, hereinafter called 3D variables. From the analysis of the dynamic pressure patterns, the following 12 regional loading parameters (named LOAD variables) were calculated: peak pressure (PP), maximum pressure-time integral (PTI), and PTI normalized to contact time (PTI_N_) within each of the four forefoot regions, i.e. HLX, I MET, II-IV METs and V MET. Two functional (FUNC) variables were calculated, i.e. the contact time (CT) and the arch index (AI); the latter is the ratio between the footprint area of the midfoot and that of the whole foot without toes. Two biological (BIOL) variables were taken, age and BMI. Two clinical (CLIN) variables were also analyzed, years of disease (YOD) and an original neuropathy-related variable, i.e. the Neuropathy Score and Vibration Perception Threshold (NS-VPT). This is expressed in relative units since it is the sum of four contributions [26], each one divided by the corresponding max value. In detail, the following four contributions were taken: the MNSI score from patient’s list of questions about history (max score 15); the MNSI score from health professional’s list of questions for Physical Assessment (max score 10); the VPT at hallux (max value 40 Volt); the VPT at malleolus (max value 40 Volt).

Correlations were performed among all groups of variables, for the whole set of patients (ALL) as well as separately for the patient groups N and D, and here reported in terms of coefficient of determination R^2^ (Pearson’s correlation analysis implemented through R3.4.3 software version© The R Foundation). 3D versus LOAD, FUNC, BIOL, and CLIN variables resulted in 1512 inter-variable correlations. Intra-variable analysis implied 3486 correlations within 3D variables and 66 within LOAD variables, for a total of 3552 correlations. These were performed for each group and were meant to explore respectively correlations between 3D variables and all the other analysed variables, and possible dependencies among 3D and among LOAD variables. Only significant inter-variable correlations (*p* < 0.05) were discussed and interpreted.

## Results

Demographic and clinical information of the 16 patients analysed, together with other major clinical parameters are reported in Table 1.

**Table 1:** Demographic and clinical information of the patients analyzed

	n	Age (years)	BMI (kg/cm2)	YOD (years)	NS-VPT ^a^	CT (ms)	AI ^a^
Neuropathic (N)	7 (7 M/0F)	58.1 ± 15.6	25.7 ± 2.0*	35.0 ± 11.5	2.53 ± 0.46	709 ± 104	0.25 ± 0.03
Diabetes only (D)	8 (1 M/7F)	46.2 ± 17.2	22.6 ± 2.5	30.8 ± 15.8	0.97 ± 0.34	646 ± 62	0.21 ± 0.06
ALL (N + D + LADA)	16 (9 M/7F)	51.8 ± 16.5	24.0 ± 2.6	31.3 ± 14.4	1.70 ± 0.86	680 ± 86	0.22 ± 0.05
LADA	1 (M)	51.3	23.7	9.3	1.66	740	0.15

Legend: the major demographic and clinical information of the patients analysed (Mean ± standard deviation). Statistically significant differences from the corresponding value in D (Student’s t-test between N and D, *p* < 0.05) are denoted with *.

^a^NS-VPT and AI are expressed in a relative unit: the former is the sum of four contributions (MNSI-history, MNSI-physical assessment, VPT at hallux, VPT at malleolus), each one divided by its corresponding full scale; the latter is calculated as the ratio between the midfoot area of the footprint and the whole footprint area without toes

**Fig. 4:**
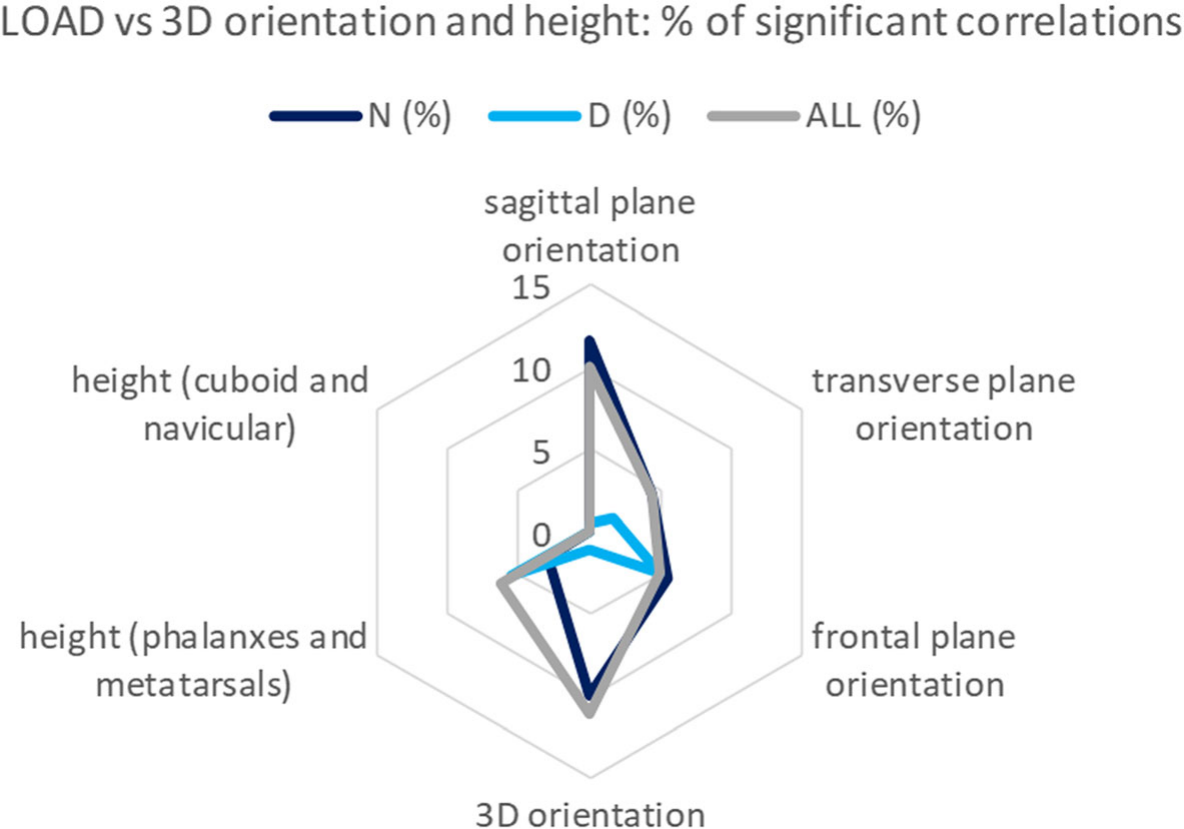
Radar plot of the number of significant correlations. Radar plot of the number of significant correlations (%) between dynamic plantar loading (the LOAD variables PP, PTI and PTI_N_) at the four forefoot regions (HLX, I MET, II-IV METs and V MET) and all the 3D variables. The latter are grouped as angles in the lateral (180 correlations per patients’ group), transverse (180 correlations), and frontal (180 correlations) planes, and in 3D (180 correlations), height of forefoot (phalanxes and metatarsals, 240 correlations) and of midfoot (cuboid and navicular, 48 correlations) bones. Correlations are plotted for the group of all patients (ALL) and for the neuropathic (N) and non-neuropathic (D) type 1 subgroups

In total, 244 significant correlations (*p* < 0.05) were found (106 within group ALL, 85 within N, 53 within D), 160 of which between LOAD and 3D variables (69, 64 and 27 within the three groups, respectively). A summary of the latter (Fig. 4) revealed that bone inclinations in the lateral plane and in 3D have the highest percentages of significant correlations with LOAD (10 and 11% respectively), and therefore the present preliminary analysis focused on these angles. Correlations were weak-to-moderate in group ALL (median R^2^ [Q1-Q3 interquartile range]: 0.29 [0.27–0.32], 37 significant correlations, 10%) and moderate-to-strong in group N (0.68 [0.60–0.74], 39 significant correlations (11%)). Only two significant correlations, though moderate, were found in group D, both with R^2^ = 0.62, strongly correlated between them.

**Fig. 5:**
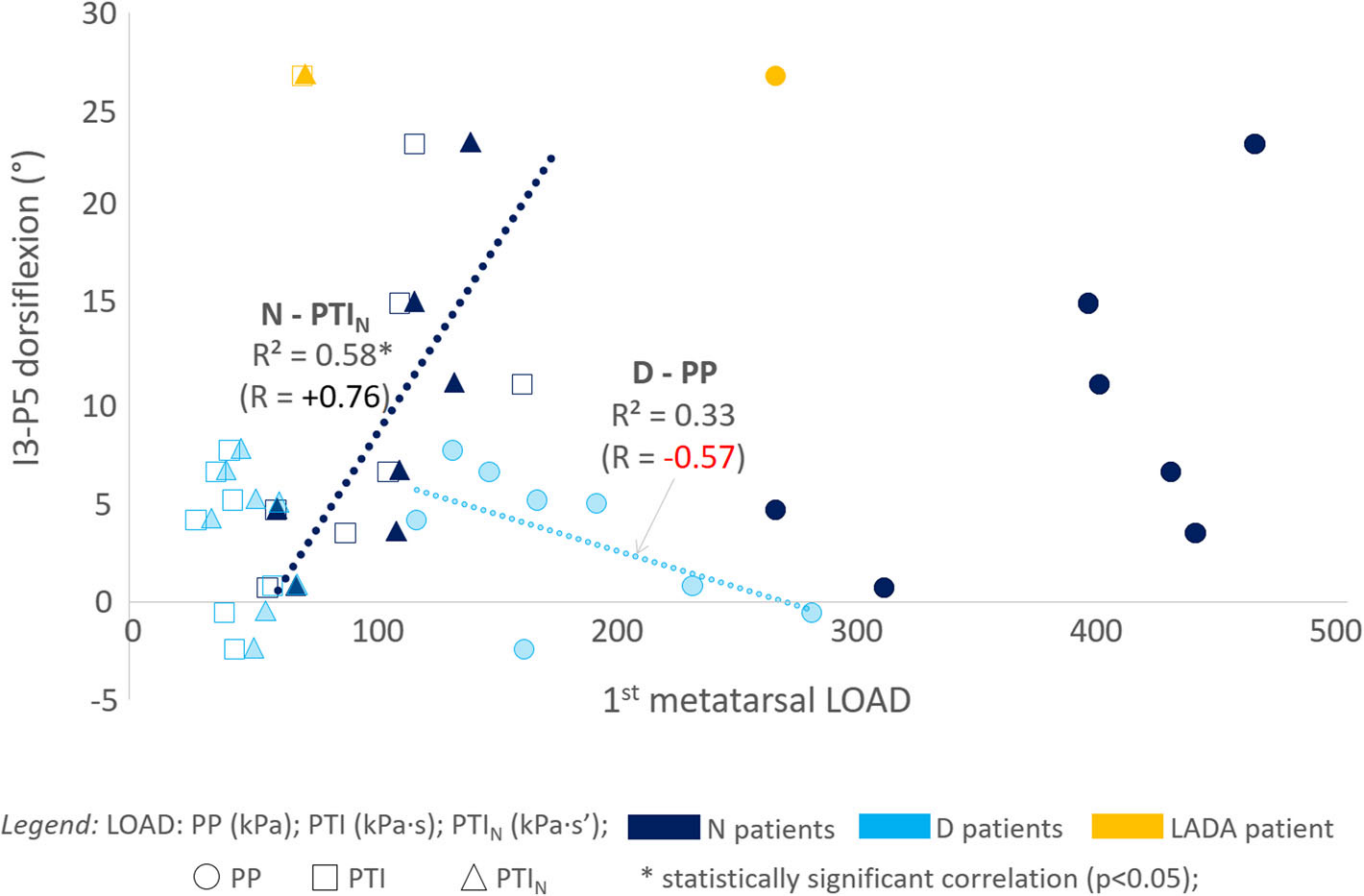
Scatter plot for a phalanx 3D inclination with respect to LOAD changes. Scatter plot for the 3D inclination angle, i.e. dorsiflexion of the 5th phalanx (I3-P5), with respect to changes of the LOAD variables (PP, PTI and PTI_N_) at the I metatarsal region for N (dark blue) and D (light blue) sub-groups separately, and for the LADA patient (orange). Within each group, the regression line is drawn for the LOAD variable with the highest correlation, i.e. PTI_N_ in N and PP in D

LOAD variables PP, PTI and PTI_N_ referred to the same foot region were strongly correlated among them; none of them performed better than the others in all cases, rather they complemented each other. Thus, for each bone segment and pathologic group, the highest correlation (R^2^) was reported in the following analysis and plots. LOAD under the 5th metatarsal never showed significant correlation with 3D variables. Conversely, LOAD under the central metatarsals’ region showed moderate-to-strong correlation with dorsiflexion at the 2nd and 3rd phalanxes, in ALL (R^2^ 0.43) and N (0.77) groups. LOAD under the 1st metatarsal showed weak correlation in ALL (0.30), likely because of opposite trends in the two subgroups (Fig. 5): in N it showed strong and independent correlation with 3rd to 5th phalanx dorsiflexion (0.64, 0.85, 0.73, respectively); in D it showed a general trend to correlate with plantarflexion at the 5th phalanx, though not significant. LOAD at the hallux correlated with plantarflexion at the 3rd phalanx in ALL (0.31), at the 1st phalanx in N (0.59) and at the 5th phalanx in D (0.62).

**Fig. 6:**
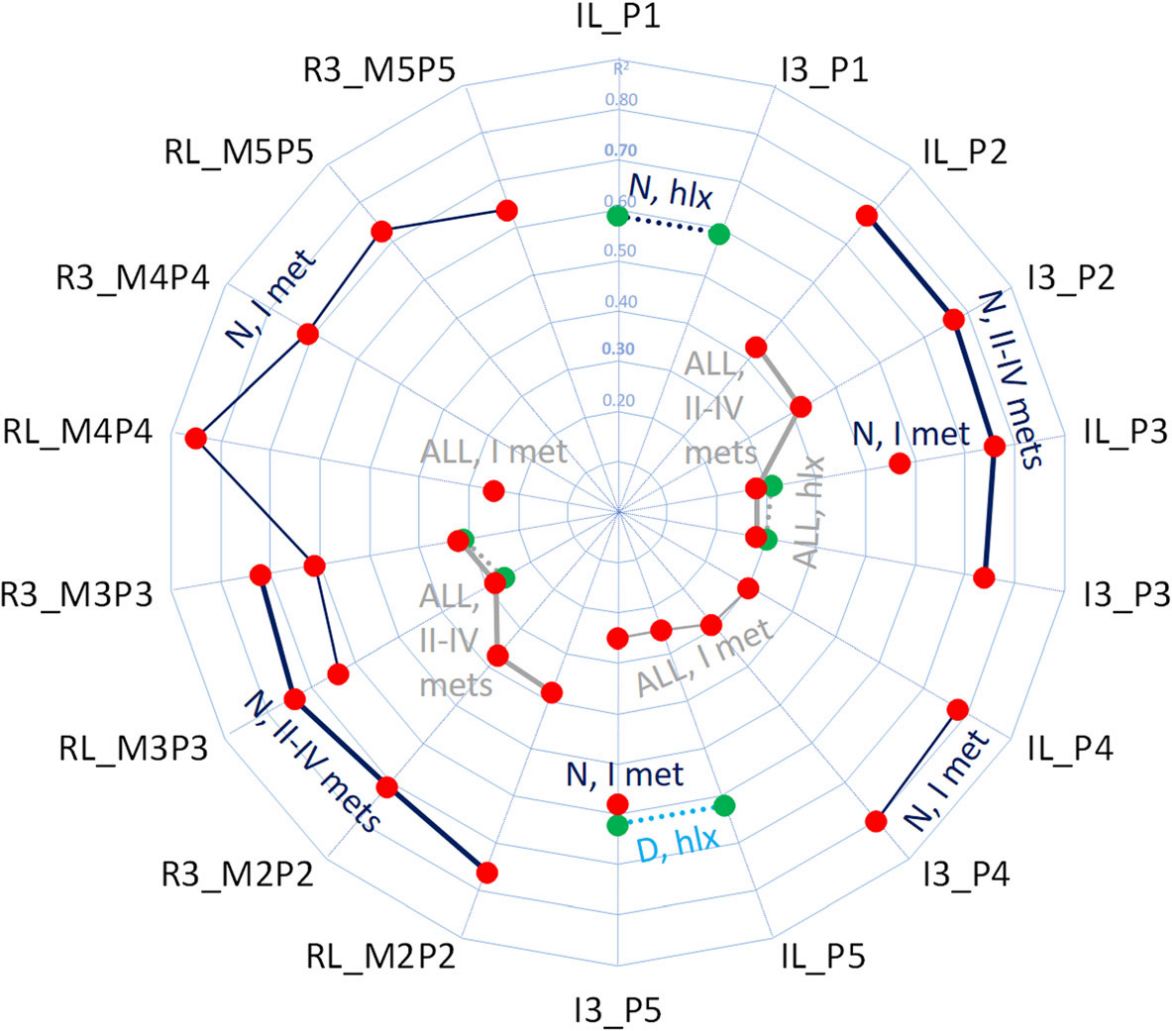
Radar plot of correlations between LOAD and 3D variables. Radar plot of the Pearson coefficients (R^2^) between LOAD and those 3D variables found significantly (*p* < 0.05) correlated. Corresponding lateral and 3D angles are reported sequentially to better detect relevant similar figures. LOAD correlations with dorsiflexion are marked in red, with plantarflexion in green. Correlations are referred to either the whole sample of patients (ALL, in grey), or to N (dark blue) or D (light blue) sub-groups. Line segments are used to highlight adjacent relevant results for the same variable of the same group: dotted lines represent the HLX region, thin solid lines represent the I MET region, and thick solid lines represent the II-IV METs region (V MET region did not show significant correlations). Correlations with R^2^ up to 0.30 were interpreted as weak, in between 0.30 and 0.70 as moderate, above 0.70 as strong

A thorough radar plot for R^2^ (Fig. 6) reports all significant correlations between LOAD variables and angles in the lateral plane and in 3D. Clear associated variables with strong correlation with LOAD can be detected between IL and I3 variables, for 2nd and 3rd phalanges, top-right zone of the plot, and between RL and R3 for the 2nd and 3rd metatarso-phalangeal joints, bottom-left of the plot. Associations between RL and R3 were also found, for 4th and 5th metatarso-phalangeal joints, top-left of the plot, though for another loading region. These three series, however, were found only in the N sub-group. Other associations were found for ALL and D sub-groups, central part of the plot, though by less strong correlations. Values of the significantly correlated parameters are reported in Table 2.

**Table 2:** Mean ± standard deviation of LOAD parameters, and of those 3D alignment parameters which significantly correlated (Pearson’s correlation test) with them

	Neuropathic patients (N)	Non-neuropathic patients (D)	All patients (ALL)	*Patient with LADA*
**Load at Hallux**				
PP (kPa)	336 ± 116	396 ± 153	363 ± 133	*290*
PTI (kPa*s)	69 ± 39	80 ± 36	74 ± 35	*61*
PTI_N_	71 ± 36	93 ± 42	82 ± 39	*62*
**Load at I metatarsal head**				
PP (kPa)	391 ± 73^a^	182 ± 55	279 ± 120	*270*
PTI (kPa*s)	102 ± 37^b^	44 ± 11	71 ± 38	*72*
PTI_N_	107 ± 31^c^	51 ± 12	77 ± 35	*73*
**Load at II-IV metatarsal heads**				
PP (kPa)	477 ± 216	375 ± 146	418 ± 178	*355*
PTI (kPa*s)	131 ± 47^d^	85 ± 31	106 ± 43	*96*
PTI_N_	143 ± 63	98 ± 36	118 ± 52	*97*
**Load at V metatarsal head**				
PP (kPa)	283 ± 206	172 ± 108	220 ± 160	*165*
PTI (kPa*s)	93 ± 66	53 ± 34	70 ± 53	*38*
PTI_N_	98 ± 68	61 ± 39	76 ± 54	*39*
**Absolute 3D dorsiflexion of phalanx**				
I3_P1 (°)	−9.6 ± 3.1	−11.4 ± 4.4	−10.8 ± 3.9	*−14.7*
I3_P2 (°)	4.9 ± 9.8	5.8 ± 9.1	5.7 ± 8.9	*10.0*
I3_P3 (°)	5.7 ± 8.2	2.6 ± 7.8	4.6 ± 7.9	*12.8*
I3_P4 (°)	7.2 ± 6.8	1.7 ± 7.6	5.3 ± 8.4	*21.3*
I3_P5 (°)	9.4 ± 7.9	3.4 ± 3.6	7.5 ± 8.1	*26.9*
**Relative (metatarsal-phalanx) lateral dorsiflexion**				
RL_M1P1 (°)	11.7 ± 6.0	12.8 ± 4.8	12.2 ± 5.1	*11.7*
RL_M2P2 (°)	30.4 ± 10.9	32.2 ± 7.0	32.1 ± 9.0	*43.4*
RL_M3P3 (°)	27.9 ± 9.9	24.2 ± 6.7	26.8 ± 8.6	*39.4*
RL_M4P4 (°)	21.5 ± 7.4	16.9 ± 6.1	20.4 ± 8.7	*41.5*
RL_M5P5 (°)	16.1 ± 8.4	12.9 ± 4.3	16.1 ± 9.1	*40.8*

Legend:

^a^*N* statistically higher than D (Student’s t-test, *p*-value < 0.0001)

^b^*N* statistically higher than D (Student’s t-test, *p*-value = 0.0052)

^c^*N* statistically higher than D (Student’s t-test, *p*-value = 0.0024)

^d^*N* statistically higher than D (Student’s t-test, *p*-value = 0.0494)

Last column on the right contains the corresponding values for the only one LADA patient included in the dataset. Negative Dorsiflexion means Plantarflexion.

LOAD at Hallux correlated with 3D phalanx absolute inclination I3_P1 (N: R^*2*^ = 0.59; *p*-value = 0.045), I3_P3 (ALL: R^*2*^ = 0.30; *p*-value = 0.030) and I3_P5 (D: R^2^ = 0.62; *p*-value = 0.020).

LOAD at I metatarsal head correlated with 3D phalanx absolute inclination I3_P4 (N: R^*2*^ = 0.80; *p*-value = 0.007; ALL: R^*2*^ = 0.29; *p*-value = 0.030) and I3_P5 (N: R^2^ = 0.58; *p*-value = 0.047; ALL: R^*2*^ = 0.25; *p*-value = 0.048), and with lateral metatarsal-phalanx relative orientation RL_M3P3 (N: R^*2*^ = 0.64; *p*-value = 0.030), RL_M4P4 (N: R^2^ = 0.85; *p*-value = 0.003; ALL: R^*2*^ = 0.25; *p*-value = 0.049) and RL_M5P5 (N: R^*2*^ = 0.73; *p*-value = 0.014).

LOAD at II-IV metatarsal heads correlated with 3D phalanx absolute inclination I3_P2 (N: R^*2*^ = 0.77; *p*-value = 0.010; ALL: R^*2*^ = 0.42; *p*-value = 0.007) and I3_P3 (N: R^2^ = 0.74; *p*-value = 0.013; ALL: R^*2*^ = 0.28; *p*-value = 0.037), and with lateral metatarsal-phalanx relative orientation RL_M2P2 (N: R^*2*^ = 0.76; *p*-value = 0.011; ALL: R^2^ = 0.38; *p*-value = 0.011) and RL_M3P3 (N: R^*2*^ = 0.74; *p*-value = 0.013; ALL: R^*2*^ = 0.28; *p*-value = 0.034).

LOAD at V metatarsal head did not correlate with 3D bone alignment parameters

As for the two FUNC variables, AI negatively correlated with 1st phalanx dorsiflexion in ALL (0.60) and in D (0.80), whereas CT showed strong negative correlation in D only, with 2nd and 3rd metatarsals plantarflexion (0.63) and with 4th phalanx dorsiflexion (0.87). No significant correlations were found with BIOL variables, with the only exception of weak negative correlation between AGE and 4th phalanx dorsiflexion in ALL (0.31). Finally, CLIN did not correlate in terms of YOD, whereas the combined NS-VPT variable showed strong correlation only with 2nd and 3rd phalanxes dorsiflexion in N (0.78).

## Discussion

The foot skeleton is a very complex structure that changes according to a number of pathological and physiological conditions, including the critical diabetic foot. Bone alignments also change considerably during the course of disease, and from non-weight-bearing to weight-bearing condition; only the latter offers a realistic representation of this structure during daily living activities. With the modern CBCT devices, quantification of 3D bone absolute and relative alignments is now possible in upright single- or double- leg weight-bearing postures, minimizing errors and artefacts also due to operator-dependent identification of anatomical references. This technique overcomes previous planar views under load in standard X-ray, or 3D views in unloaded conditions, i.e. supine position, in standard computed tomography. This also enables careful angle measurements in each anatomical plane of the foot, and also in 3D exactly along the plane of the longitudinal axis of the bone [22]. These CBCT scans also allow for possible new measures [8, 27], particularly at the forefoot [12], and have potential for thorough biomechanical analyses in physiological and pathological feet. It is worth mentioning that angular measurements in lateral views, as traditionally obtained from standard radiographs, can be different from more realistic 3D angles, which are not affected by positioning and deformities of the foot [22]. The present techniques are expected to be valuable for many other applications in biomechanics and also for foot and ankle treatments. The availability of 3D spatial models of foot bones in weight-bearing also allows calculation of other clinically relevant measurements, based for example on mid-diaphyseal axes or line segments between anatomical landmarks.

The present study thus aimed at correlating, for the first time, these 3D bone architecture measurements from CBCT scans in static weight-bearing with corresponding dynamic plantar pressure measurements. The combination of the two analyses in patients with type 1 diabetes proved to be valuable, revealing meaningful loading pattern alterations associated to structural changes of the foot bone architecture in patients with neuropathy. Interestingly, the only patient with a different clinical diagnosis from type 1 diabetes (the LADA patient), showed an association between bone alignment and plantar load which was different either from neuropathic or non-neuropathic type 1 Diabetes patients (Fig. 5). This is not an evidence, but may promote future investigations on the specificity of the present combined biomechanical and functional assessments.

These original measures seem particularly relevant for the diabetic foot. In particular their combination with established plantar loading measurements in dynamic conditions can provide fundamental insights for thorough assessments of the frequent complications at the foot, both in larger populations and in single patients for specific problems. The diabetic foot progressively shows combined complex changes in muscle structure and function such as the reduction of the cross-sectional area, mass and strength, conduction velocity and motor units, as well as changes in the microstructure and biochemical properties not only at the muscles but also at soft tissues. A proper personalized diagnosis based on these parameters would imply invasive analyses, high costs, and difficult overall evaluations. On the other hand, the present integration of CBCT-based measures in the usual biomechanical assessment of the diabetic foot might help detecting relevant foot changes well in advance of clinical problems, indicative of the final effect of all those complex diagnostic parameters. This would definitely support personalized and optimized prevention of the diabetic foot, as well as the design and the assessment of more effective orthotics or surgical interventions.

This exploratory investigation showed associations between load and inclination and orientation of the forefoot bones, in the lateral plane and in 3D more than in other anatomical planes, especially in ALL and N groups. More specifically, association was found with respect to the absolute dorsiflexion of the phalanxes, and with relative phalanx-to-metatarsal angles. Other linear 3D bone variables, among which midfoot and forefoot heights either absolute or relative, showed poor or none correlation with LOAD, particularly in N group: while navicular and cuboid heights never correlated, 6% of weak correlations (R^2^: 0.26–0.29) were found in ALL group (4% with metatarsals M2-M5, 2% with phalanx P3), 3% of moderate correlations (R^2^: 0.58–0.61) were found in N group but only with metatarsals M2 and M4, and 5% of moderate-to-strong correlations (R^2^: 0.56–0.70) were found in D group (1% with metatarsals M2 and M5, 4% with phalanxes P2 and P5). Whether this finding is due to the present patient population, to the measurement and processing techniques, to the physiological high variability of these parameters, or to other reasons should be the topic for future investigations.

There are several limitations in this study and these techniques. The sample size is small, and larger populations in future studies would be recommended for more robust evidence. However, the radiations and the combinations with dynamic plantar loading data are definitely critical constraints in this respect. Neuropathic patients were all males, whereas non-neuropathic were seven females and one male; this was not for the inclusion criteria, but was the result of the open population recruitment based on the outpatient clinical service. With the present CBCT scanner, the field of measurement is limited, and large-size feet cannot be scanned entirely; however, as in the present study, the anatomical area of major interest, i.e. the forefoot, can be targeted easily for complete data collection during the scans. It was not possible during CBCT scans to measure the exact amount of weight on the foot; however, the patients were all instructed in the same way and encouraged to load the foot as much as possible. Another relevant issue in CBCT scanning, though less critical, is the overall position and inclination of the leg, which is associated to the overall posture and in particular the inclination and axial rotation of the pelvis and trunk; this may even result in different architectures of the foot bones. Artifacts due to deformed feet or abnormal amount of soft tissues might also potentially represent a relevant issue in any diabetic population; in this exploratory study, however, only type 1 adult patients with diabetes with normal or slight overweight BMI and limited deformity were analyzed, which likely led to statistically significant correlations even in the present small samples. The procedure for the calculation on bone and joint angles is quite complex, and thus time consuming, in addition to be invasive; in the perspective of possible future routine exploitations it should be considerably simplified. In addition, by focusing only on those few bones of interest, the procedure might be further simplified. Finally, the present measurements for the 3D variables do not have a control reference yet; this may be available in the future, but the definition of normality in this context, and the access to computer tomography of healthy volunteers would not be easy. The feet analyzed were not ulcerated, thus the relevant risks cannot be assessed or derived from the present results. Further studies are however necessary to establish whether the new insight counterbalances the complexity and resources associated with the present novel integrated approach.

## Conclusions

Statistically significant correlations were found between CBCT-based measurements of static 3D foot bone alignments in weight-bearing and corresponding loading patterns from dynamic gait measurements of plantar pressure, in type 1 diabetes separately for neuropathic or non-neuropathic patients. Peak pressures under metatarsal bones were not necessarily justified by bone and joint deformities on those corresponding rays. These original measures of foot bone structure were also found to correlate with arch index, contact time, age and with an original neuropathy-related variable.

## List of abbreviations

CBCT: Cone-beam computed tomography
PCA: Principal Component Analysis
3D: Three-dimensional
2D: Bi-dimensional
MNSI: Michigan Neuropathy Screening Instrument

ANGLES/BONES (3D variables), made of four characters:

First:

“I”: Denotes the absolute inclination angles of a single bone segment
“R”: The relative orientation between two bone segments

Second:

“3”: For the 3D, “L” “F” and “T” respectively for the lateral, frontal and transverse planes
“Hg”: The height of the bone segment

Third and fourth:

“P”: The five phalanxes, from 1 to 5
“M”: The five metatarsals, from 1 to 5
“I3_P5”: Reports the 3D absolute inclination angle of the 5th phalanx

GROUPS:

D: Diabetic patients without neuropathy
N: Diabetic patients with neuropathy
LADA: Patient with latent autoimmune Diabetes of the adults
“ALL”: All together in the group

LOAD variables:

PP: Peak pressure
PTI: Pressure-time integral
PTI_N_: Pressure-time integral PTI normalized to contact time

FOREFOOT regions:

HLX: Under the hallux
I MET: Under the I metatarsal head
II-to-IV METs: Under the II-to-IV metatarsal heads
V MET: Under the V metatarsal head

FUNCtional [CT, AI], BIOLogical [BMI], and CLINical [YOD, NS-VPT] variables:

CT: Contact time
AI: Arch index
BMI: Body mass index
YOD: Years of disease
NS-VPT: Neuropathy Score and Vibration Perception Threshold

## Data Availability

The datasets used and/or analysed during the current study are available from the corresponding author on reasonable request.
